# Integrating elastic band into physical education classes to enhance strength training

**DOI:** 10.3389/fpsyg.2023.1037736

**Published:** 2023-02-15

**Authors:** Qun Fang, Xiaochao Zhang, Yuhang Xia, Fang Huang

**Affiliations:** School of Physical Education, Qingdao University, Qingdao, China

**Keywords:** elastic band, strength training, physical education, critical period, motor development

## Introduction

Physical education (PE) is considered one of the most universally applicable and effective strategies for health and physical activity promotion in children and adolescents (Bukowsky et al., 2014). By reviewing the effects and outcomes of PE, researchers identified benefits in the development of fundamental movement skills, physical competences, social skills and behaviors, self-esteem, physical and mental health, cognitive performance, and academic achievement (Bailey, 2006; Donnelly et al., 2016). PE nowadays is characterized by a comprehensive physical activity and health promotion program which is more than activity-only classes. Instead, an increasing attention is paid to essential components such as conceptual PE which teaches concepts and principles of physical activity *via* text materials and classroom sessions (Corbin, 2021) and PE homework which increases physical activity after school (Huang et al., 2022). Current trends in PE indicate a growing need to incorporate different types of fitness activities for balanced motor development (Lubans et al., 2010). However, a thorough examination on the existing physical activity guidelines worldwide indicated particular interests in aerobic training and cardiovascular fitness, but a lack of emphasis on resistance exercise (Myer et al., 2015). The researchers thus called for increasing attention to the qualitative aspects of program design rather than the mere focus on quantity of daily physical activity.

Benefits of strength training for children and adolescents include improvements in health, fitness, injury prevention and rehabilitation, and physical literacy (Faigenbaum and Myer, 2010; Stricker et al., 2020). Research evidence has shown significant influences of strength training on physical activity behaviors. In a study involving 102 school children, the resistance training program which developed upon seven basic barbell exercises induced significant increase in daily spontaneous physical activity behavior, with the less active children indicating the greater increase (Meinhardt et al., 2013). The results suggest strength training an important motivation for children to become physically active.

Strength training for children and adolescents can also be justified by a developmental perspective. The rate of motor development varies across the lifespan. The window during which brain connections are more plastic and receptive to the stimulus is known as the critical period (Gabbard, 2012). People experience a number of critical periods for motor development in childhood and adolescence (Gale et al., 2004). Adequate stimulus during the critical periods optimizes improvement for a specific function. As the nervous system becomes less plastic in maturation, missing the window of opportunity makes an individual difficult to reach his/her full potential (Salkind, 2002). The timing of brain development indicates that preadolescence and early adolescence are critical periods for developing muscular strength (Gogtay et al., 2004; Myer et al., 2011; Curlik and Shors, 2013).

Overload is an important consideration in program design for strength training. The training effects can be largely limited due to inadequate load stimulus (Haff and Triplett, 2015). Traditional strength training requires various equipment such as dumbbell, barbell, and weight machines to provide external load. However, a large space is needed for the heavy and stationary equipment (Bergquist et al., 2018). Practical concerns may arise for considerations of space, convenience, and safety under PE settings. The space concern lies in the fact that elementary and middle schools, particularly in Asian countries, are characterized by small campus and limited classroom space (Johns and Ha, 1999). Allocating a specific room for strength and conditioning class may be difficult to put into practice. Even a classroom could be assigned, the equipment may be inadequate for a large class size over thirty or forty students. More importantly, safety concerns may prevent practitioners from giving a strength and conditioning class if one physical education teacher has to take care of the whole class. Students can be exposed to risks of injury due to inappropriate use of equipment, too much load in exercise, and improper movement skills. Therefore, a practical instrument to manage exercise load can enrich options for PE teachers to design and implement strength training classes.

The importance of developing muscular strength during the critical period and the challenges in pedagogical practice demand an effective solution to integrate strength training into PE classes. An alternative instrument for strength training is elastic band which has gained an increasing attention from researchers and practitioners. Elastic band training has been considered a safe and progressive overload technique with practical advantages of low cost, simplicity, versatility, and portability over traditional strength training (Mascarin et al., 2017; Aloui et al., 2019; Hammami et al., 2022). The current study proposed the opinion that elastic band is a promising instrument to implement strength training in PE settings. To substantiate the opinion, we searched for potential studies which applied elastic band to PE classes. However, direct information is unavailable due to the lack of the research which applied elastic band exercise to PE classes. Relevant evidence for the program design was summarized from the research on young athletes. The following sections firstly indicated the integrative characteristics of elastic band for various training modes. Further analysis focused on the elastic band protocols regarding volume, frequency, and duration. Based on the considerations from both research and practice, we discussed the feasibility of integrating elastic band into PE settings to facilitate strength development for students.

## Integrative characteristics of elastic band

Elastic band produces variable resistance with increasing load during stretching and maximum tension at full extension of the band (Stevenson et al., 2010). The variable loading through the entire range of motion allows athletes to optimally use mechanical advantage and overcome greater mechanical disadvantages at different angles (Ebben and Jensen, 2002). Variable resistance training has been considered an effective training method for muscular strength (Suchomel et al., 2018). Similar electromyographic (EMG) patterns have been identified in elastic band training compared with machine (Aboodarda et al., 2011; Jakobsen et al., 2012) and free-weight training (Bergquist et al., 2018). Additionally, research findings reported comparable effects between elastic band and traditional resistance training (Andersen et al., 2010; Melchiorri and Rainoldi, 2011). It is also worth noting the increased stability requirement during elastic band training. The changing forces give rise to an unstable condition which induces higher neural drive and greater activation of muscle groups to maintain a stable posture (Saeterbakken et al., 2011). Compared with the strength training under a stable condition, elastic band may result in greater energy expenditure in exercise (Bergquist et al., 2018).

Another characteristic of elastic band is to effectively enhance eccentric stimulus of training given that the external load is higher in the initial part of the eccentric phase (Israetel et al., 2010). Compared with concentric and traditional training, eccentric training stimulates neuromuscular adaptations such as fast motor unit recruitment and high firing rate (Suchomel et al., 2018). Accordingly, vertical jump, sprint, and change of direction depending on the neuromuscular functions can be benefited by eccentric training. Eccentric strengthening of elastic band is evident in a hip-adductor training for soccer players (Jensen et al., 2014). After 8 weeks of elastic band intervention, the participants indicated larger increase in eccentric hip-adductor strength than the counterparts only performing soccer training. Another study involving young volleyball players reported significant improvement in countermovement jump and broad jump after an 8-week elastic band training program. The magnitude of improvement was greater in the elastic band training group compared with the control group which only performed the regular volleyball training (Hammami et al., 2022). In addition, applying elastic band to lower limb training (knee extension and hip extension) has indicated positive effects on change of direction for young handball players. Participants receiving the elastic band training outperformed their counterparts receiving standard handball training in the change of direction test after the intervention program. The significant interaction effect between group and time indicated larger improvement associated with the elastic band training (Aloui et al., 2019, 2020).

The characteristics of plyometric training are also represented in the elastic band training. Plyometric training takes advantage of the stretch-shortening cycle, which involves a rapid concentric contraction following an eccentric muscle action (Suchomel et al., 2018). Combining push-up with elastic band is effective for plyometric training in which the band is placed across shoulder blades. The eccentric phase of lowering the body stretches the band which increases load to the concentric phase of propelling up the body. The loaded concentric action has shown particular effects on power and rate of force development. Research provided evidence that the plyometric training protocol of push-up with elastic band induced significant improvement in upper limb power and sport specific performance such as ball throwing velocity (Aloui et al., 2021).

Providing load during stretching exercise makes elastic band an appropriate instrument for flexibility training. A 5-week elastic band training for rugby players indicated significant improvement in lower limb flexibility (Guillot et al., 2019). Particularly, the greater joint range of motion (ROM) was evidenced by 2.31% increase in sit-and-reach and 29.16% increase in side split. In addition to the greater joint ROM, elastic band training was proved to enhance joint stability. Lister et al. (2007) implemented an elastic band training for college athletes. Based on EMG measures, the researchers identified increased shoulder muscular activity in upper trapezius, lower trapezius, and serratus anterior after elastic band training compared with traditional resistance technique using cuff weight. The increased scapular activity suggests enhanced shoulder stability which is important for functional mobility and injury prevention.

The existing literature suggests elastic band an integrative training method which includes variable resistance training, eccentric training, plyometric training, and flexibility training. The combined characteristics enhance multiple physical abilities such as strength, power, and change of direction. Therefore, it is reasonable to use elastic band to assist in development of strength, power, and flexibility in PE classes.

## Class design based on elastic band exercise

One of the common applications in strength and conditioning training is to attach the elastic band to free weight equipment (i.e., barbell and dumbbell) for additional load (Shoepe et al., 2011). In this case, the load is the sum of the weight and the resistance of the stretched elastic band (Lorenz, 2014). The increased load has been reported to enhance strength and power gains compared with the free weight training alone (Wallace et al., 2006; Rivière et al., 2017). Strength exercise in PE is usually implemented by body weight exercise. While body weight exercise (e.g., push-ups, squats, and sit-ups) is convenient to perform in class settings, the limited load stimulus is not adequate for maximal strength development given that simply increasing repetitions and volume leads to particular effects on muscular endurance (Suchomel et al., 2018). Elastic band offers a feasible solution to the overload issue in PE classes. Body weight exercise plus elastic band can be effective for maximal strength adaptations.

Recent studies provided supportive evidence for the positive effects of combining elastic band with the body weight exercises on muscular strength. A training protocol applied elastic band to hamstring curl exercise for young basketball players (Kamandulis et al., 2020). Significant improvement was identified in knee extension and flexion tests, indicating the positive effects of the elastic band training on lower limb strength. There is also evidence for elastic band training developing upper limb strength. In a 9-week intervention which integrated elastic band training into regular handball training, significant improvement in the maximal isometric strength of the internal rotator was reported in the elastic training group compared with the regular handball training group (Bauer et al., 2021).

The existing studies usually adopted 8 weeks of elastic band training (Jensen et al., 2014; Aloui et al., 2019, 2021; Hammami et al., 2022). In addition, protocols with a shorter duration (5 or 6 weeks) also reported significant improvement in upper and lower limb strength (Thorborg et al., 2016; Mascarin et al., 2017; Rivière et al., 2017; Kamandulis et al., 2020). The protocol durations are within the period of a full semester. It is reasonable to expect positive effects of elastic band exercise under PE settings. Training load is another primary consideration in class design. Due to the large variability of the students' physical abilities, a feasible approach to determine the training load is to use the relative resistance. In a 6-week program, the relative load was 15 repetition maximum (RM) in week 1, and then progressed to 10 RM during weeks 2–4 and to 8 RM during weeks 5–6 (Thorborg et al., 2016). Another study implemented an 8-week intervention during which a relative load of 15 ± 2 RM was applied in weeks 1–2. The load increased to 10 ± 2 RM during weeks 3–6 and to 8 ± 2 RM during weeks 7–8 (Jensen et al., 2014). PE teachers may adopt a light load (15 RM) when introducing a novel drill in class and progress to 10 RM or a higher load (8 RM) during the semester. Students usually take two to three PE classes per week, which is comparable to the frequency of training protocols in the existing studies. Despite the lack of direct information regarding the PE settings, the current studies involving young athletes still provided details as to the frequency, load, and duration of an elastic band program for students to develop muscular strength through physical education classes.

## Conclusion

The current study proposed an opinion on the promising effects of integrating elastic band training into physical education classes to promote muscular strength for students. This opinion is justified by the following reasons. First, the neurodevelopmental perspective emphasizes on the importance of developing muscular strength during the critical periods of preadolescence and early adolescence. Second, an overview on the existing literature suggests that elastic band offers integrative training modes including variable resistance training, eccentric training, plyometric training, and flexibility training. Third, research on adolescent athletes provided information as to the frequency, load, and duration of the elastic band training program. In conclusion, elastic band training can be a feasible addition to physical education classes for muscular strength development.

## Author contributions

QF and XZ prepared the draft. YX and FH worked on revision and approved the submitted version. All authors collaborated in preparing the manuscript. All authors contributed to the article and approved the submitted version.
